# Editorial: Progress on musculoskeletal disorders and stem cell therapies

**DOI:** 10.3389/fbioe.2023.1153525

**Published:** 2023-02-23

**Authors:** Feng-Juan Lyu, Songlin Peng, Jun Li

**Affiliations:** ^1^ Joint Center for Regenerative Medicine Research of South China University of Technology and The University of Western Australia, School of Medicine, South China University of Technology, Guangzhou, China; ^2^ Shenzhen Key Laboratory of Musculoskeletal Tissue Reconstruction and Function Restoration, Department of Spine Surgery and Institute for Orthopaedic Research, The Second Clinical Medical College of Jinan University (Shenzhen People’s Hospital), Shenzhen, China; ^3^ Department of Orthopaedic Surgery, University of Pennsylvania, Philadelphia, PA, United States

**Keywords:** musculoskeletal, disorders, stem cell, therapies, regeneration, bone, joint, muscle

Accompanying the aging of the population worldwide, growing concern emerges about the health and wellbeing of elderly with chronic diseases. Musculoskeletal disorders, the degenerative diseases of the musculoskeletal system, including bone, tendon, skeletal muscle, cartilage and intervertebral disc, are the leading contributors to years lived with disability worldwide, affecting people of all ages but the prevalence peaks in aged population. Musculoskeletal disorders compromise the function of bone and joints, and require extensive care and long recovery time. Musculoskeletal disorders have negative impact on the health quality of life, causing mental and physical stress to people as well as loss of work hours. Consequently, musculoskeletal disorders cause a heavy burden on patients and the whole society.

The underlying molecular mechanisms have not been fully elucidated for musculoskeletal disorders. Understanding the key factors and signaling involved in this process may greatly facilitate targeted repair and future drug development. In this Research Topic, Xiongfeng et al. reported a novel phantom-less quantitative computed tomography system, which can predict osteoporosis with relatively high accuracy and precision utilizing low-dose chest computed tomography obtained for COVID-19 screening. Zhang et al. summarized the role of adipokine signaling in osteoarthritis, which is a degenerative disease of cartilage.

Stem cells, due to their high self renewal capacity and differentiation potential, have been highlighted as a promising cell tool for tissue regeneration and engineering in musculoskeletal disorders. Mesenchymal stem cells (MSCs) (Lv et al., 2012; Lv et al., 2014) as the progenitors for mesenchymal lineages have been intensively investigated for musculoskeletal tissue regeneration (Leung et al., 2014; Chen et al., 2022; Wang et al., 2022). Other types of stem cells, such as tissue-specific progenitor cells (Lyu et al., 2019) and induced pluripotent stem cells, also received attention for their potential for tissue regeneration. In this Research Topic, Kragl et al. investigated the role of HSD11B1 in the differentiation of mesenchymal stem cells (MSCs) into adipocytes and osteoblasts, and found that HSD11B1 could increase the cortisol expression of MSCs and switch MSCs from osteogenic to adipogenic differentiation. Wang et al. discussed the recent advances in stem cell therapies for rotator cuff injuries, including bone marrow-derived MSCs, adipose-derived stem cells, tendon-derived stem cells, umbilical cord-derived MSCs, subacromial bursa-derived cells, and urine-derived stem cells. Campbell et al. discussed the ideal stem cell population for cartilage regeneration, including endogenous stem cells from cartilage, stem cell-rich dental pulp, or the adolescent growth plate, as well as MSCs from bone marrow, adipose tissue or umbilical cord, *etc.*

Stem cell-derived exosomes have independently attracted research attention for its role in targeting musculoskeletal disorders. Here, Ma et al. summarized the action mechanism of stem cell exosomes on aseptic loosening of joint prostheses, with the effects including augmenting angiogenesis, enhancing osteogenesis, suppressing osteoclast activity, and regulating immune cells and cytokines. Yuan et al. discussed the current status of exosome-based therapeutic strategy in temporomandibular joint osteoarthritis treatment, and compared the exosomes from MSCs, chondrocytes, synoviocytes, subchondral osteocytes, adipose tissue and other tissues in the expression of cell surface receptors, different contents and biological effects. They also discussed future opportunities and challenges of exosome-based treatment in temporomandibular joint osteoarthritis.

The microenvironment surrounding stem cells, including acidity, oxygen level, nutrient supply, osmolarity, etc., has non-negligible influence on the biological behavior and ultimate fate of stem cells (Huang et al., 2020). Manipulation of the local microenvironment may further enhance the regenerative effect of stem cells and benefit musculoskeletal tissue repair (Lyu, 2022). In this Research Topic, Chu et al. summarized the impact of microenvironment in stem cell-based regeneration of intervertebral disc (IVD). They discussed the recent advances on the presence of endogenous stem cells in the IVD, reviewed the impact of the microenvironment similar to IVD on the characteristics and function of MSC, summarized the current progress of IVD graft substitutes, and updated the current use of MSC transplantation for IVD diseases. Wang et al. found that the resistance of human nucleus pulposus-derived MSCs to severe acidity environment can be enhanced by Sa12b, a wasp peptide that can inhibit acid-sensitive ion channels, as demonstrated by reduced cell apoptosis, enhanced cell proliferation, chondrocyte marker expression, and stemness marker expression.

In addition to stem cell therapies, other therapies, either using various reagents or biomaterials, physiological stimulation or genetic modification, are also in the scope to regenerate damaged musculoskeletal tissue. In this Research Topic, Lin et al. reviewed the arthroscopic application of radiofrequency to treat articular cartilage lesions. They reviewed the history of radiofrequency and its application in orthopedic arthroscopy, and the underlying mechanism for the repair, in addition to the controlling factors, such as power and temperature, in ensuring the safety and effectiveness of radiofrequency therapy. Xu et al. reported the application of cell-free fat extract to prevents tail suspension–induced bone loss by inhibiting osteocyte apoptosis. Liang et al. discussed the recent progress in gene targeted therapeutic strategies in Duchenne Muscular Dystrophy.

In summary, this Research Topic highlights recent advances on the research of musculoskeletal disorders, including the degenerative mechanisms and developments of various repair strategies, including stem cell related therapies, biomaterials and others. We hope that this Research Topic will add new strength to the scientific community and contribute to future collaborations among research groups across the world.
